# The vertical topography of the carotid bifurcation – original study and review

**DOI:** 10.1007/s00276-024-03404-y

**Published:** 2024-06-07

**Authors:** Mihaela Daniela Manta, Mugurel Constantin Rusu, Sorin Hostiuc, Răzvan Costin Tudose, Bogdan Adrian Manta, Adelina Maria Jianu

**Affiliations:** 1 Department of Anatomy and Embryology, Faculty of Medicine, “Victor Babeş” University of Medicine and Pharmacy, Timișoara, 300041 Romania; 2 Division of Anatomy, Faculty of Dentistry, “Carol Davila” University of Medicine and Pharmacy, 8 Eroilor Sanitari Blvd, Bucharest, RO-050474 Romania; 3 Division of Legal Medicine and Bioethics, Faculty of Dentistry, “Carol Davila” University of Medicine and Pharmacy, Bucharest, 050474 Romania; 4 Division of Clinical Practical Skills, Faculty of Medicine, “Victor Babeş” University of Medicine and Pharmacy, Timișoara, 300041 Romania

**Keywords:** Carotid artery, Computed tomography, Anatomy, Hyoid bone, Thyroid cartilage of larynx

## Abstract

**Purpose:**

The vertical level of carotid bifurcation (CB) is commonly indicated at the superior margin of the thyroid cartilage. Few studies observed the CB vertical topography. It was aimed at studying the vertical location of the CB as referred to vertebral and anterior cervical landmarks.

**Methods:**

An archived lot of 147 computed tomography angiograms was documented for the vertical level of CB referred to vertebral and anterior cervical landmarks. The topography of the CB in relation to anterior landmarks was classified into seven types: (1) at the superior margin of the thyroid cartilage; (2) between the hyoid and the thyroid cartilage; (3) at the hyoid level; (4) between the hyoid and mandible; (5) subgonial or supragonial CB; (6) lower cervical level; (7) intrathoracic.

**Results:**

The most common locations of CB were at C3 (27.21%), C3/C4 (26.19%) and C4 (25.51%). Bilateral symmetry of CB was found in 51.7%, except for C2 and C5/C6. Type 7 was not found, type 3 occurred in 39.12%, type 2 in 24.49%, type 1 in 13.95%, type 4 in 13.61%, type 5 in 6.12%, and type 6 in 2.72% (294 CBs). Bilateral symmetry of anterior types was found in 59.86%. Statistically significant correlations were found between sex and both left and right types and vertebral levels of CB.

**Conclusions:**

The vertical topography of the CB is highly variable and has sex-related specificity. This detail should be included in the teaching of anatomy. Surgeons and interventionists should better document the carotid anatomy on a case-by-case basis.

## Introduction

The common carotid artery (CCA) ascends in the neck to divide into the external (ECA) and internal (ICA) carotid arteries. The carotid arteries have unpredictable anatomic variations. The carotid bifurcation (CB) is commonly regarded as located at the level of the upper margin of the thyroid cartilage [4, 24]. Still, it may be higher or lower than the usual levels [4].

The human anatomy texts in use provide few details on the CB variations. Bergman’s Encyclopedia of Human Anatomic Variations documented atypical vertebral levels of the CB [6].

Knowledge of the CB’s topography is essential for neck vascular surgery, radical neck dissections, carotid sinus baroreceptor stimulation, catheterisations, and aneurysms [19]. Vascular variants should be checked preoperatively.

It was therefore aimed at studying the vertical topography of the CB, which equally referred to vertebrae and anterior landmarks, such as the thyroid cartilage, hyoid bone, and the gonial angle, to check bilateral symmetry and eventual significant correlations.

## Materials and methods

There were used 150 archived angioCT files. Inclusion criteria: complete head and neck scans, no movement artefacts, and no pathological processes (tumours, massive atherosclerotic plaques, occluded arteries) distorting the carotid anatomy. Exclusion criteria: scans inadequate for observing the carotid arteries, pathological processes nearing the carotid arteries and distorting their anatomical features, previous neck surgery, hyperextension or excessive lateral rotation of the neck during the procedure, and movement artefacts. Three cases were therefore excluded. Determinations were thus made bilaterally on a retrospectively assessed lot of 147 cases, 86 males and 61 females (sex ratio = 1.4), that were scanned in the interval between 2020 and 2023.

Being a retrospective study on archived files, the informed consent was waived. The research followed principles from The Code of Ethics of the World Medical Association (Declaration of Helsinki). The responsible authorities (affiliation 1) approved the study (approval no. 45/04.09.2020).

The CTAs were performed with a 32-slice scanner (Siemens Multislice Perspective Scanner, Forcheim, Germany), with a 0.6 mm collimation and a reconstruction of 0.75 mm thickness, with 50% overlap for a multiplanar maximum intensity projection and three-dimensional volume rendering technique. The cases were documented using Horos 3.3.6 (Horos Project, Annapolis, MD, USA). Findings were verified on two-dimensional planar reconstructions and were documented with three-dimensional volume renderings.

Two classification systems of the CB were used: vertebral levels and anterior anatomical landmarks. The vertebral level of CB was studied, including the intervertebral disc levels. The vertical topography of the CB in relation to anterior landmarks, larynx, greater horn of hyoid bone and mandible was classified into seven anatomical types: Type 1 – CB at the level of the upper margin of the thyroid cartilage; Type 2 – CB in the interval between the hyoid bone and the thyroid cartilage of larynx; Type 3 – CB at the level of the hyoid bone; Type 4 – CB located in the interval between the hyoid bone and the mandible; Type 5 – CB at the level of the gonion (subgonial) or above it (supragonial); Type 6 – CB at lower cervical level; Type 7 - intrathoracic CB.

Statistical analysis was performed using SPSS v.29 for MacOS. The Pearson Chi2 test was used to assess the statistical significance of different associations. A *p*-value below 0.05 was considered significant.

## Results

The vertebral level of the CB

**Fig. 1:**
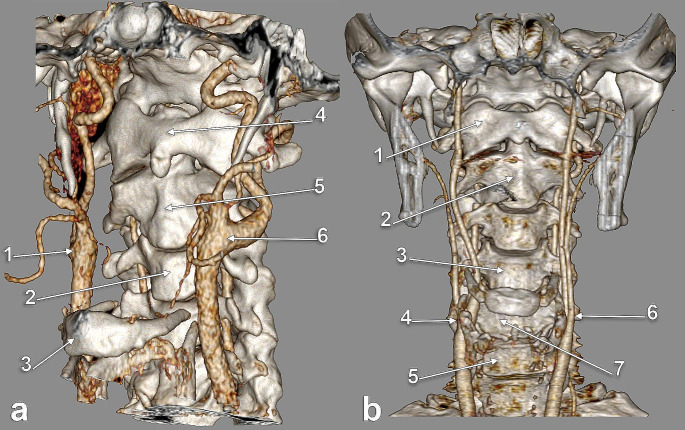
**a.** Carotid bifurcations (CB) with high location - on the left at C2 level and right at C2/C3 level. Three-dimensional volume rendering. Left anterolateral view. (1) right CB; (2) C3 vertebra; (3) hyoid body; (4) anterior arch of atlas; (5) axis; (6) left CB. **b.** Carotid bifurcations (CBs) with low localisation at the C5 level on the right side and the C5/C6 disc level on the left side. Three-dimensional volume rendering. Anterior view. (1) atlas; (2) axis; (3) C4 vertebra; (4) right CB; (5) C6 vertebra; (6) left CB; (7) C5 vertebra

**Fig. 2:**
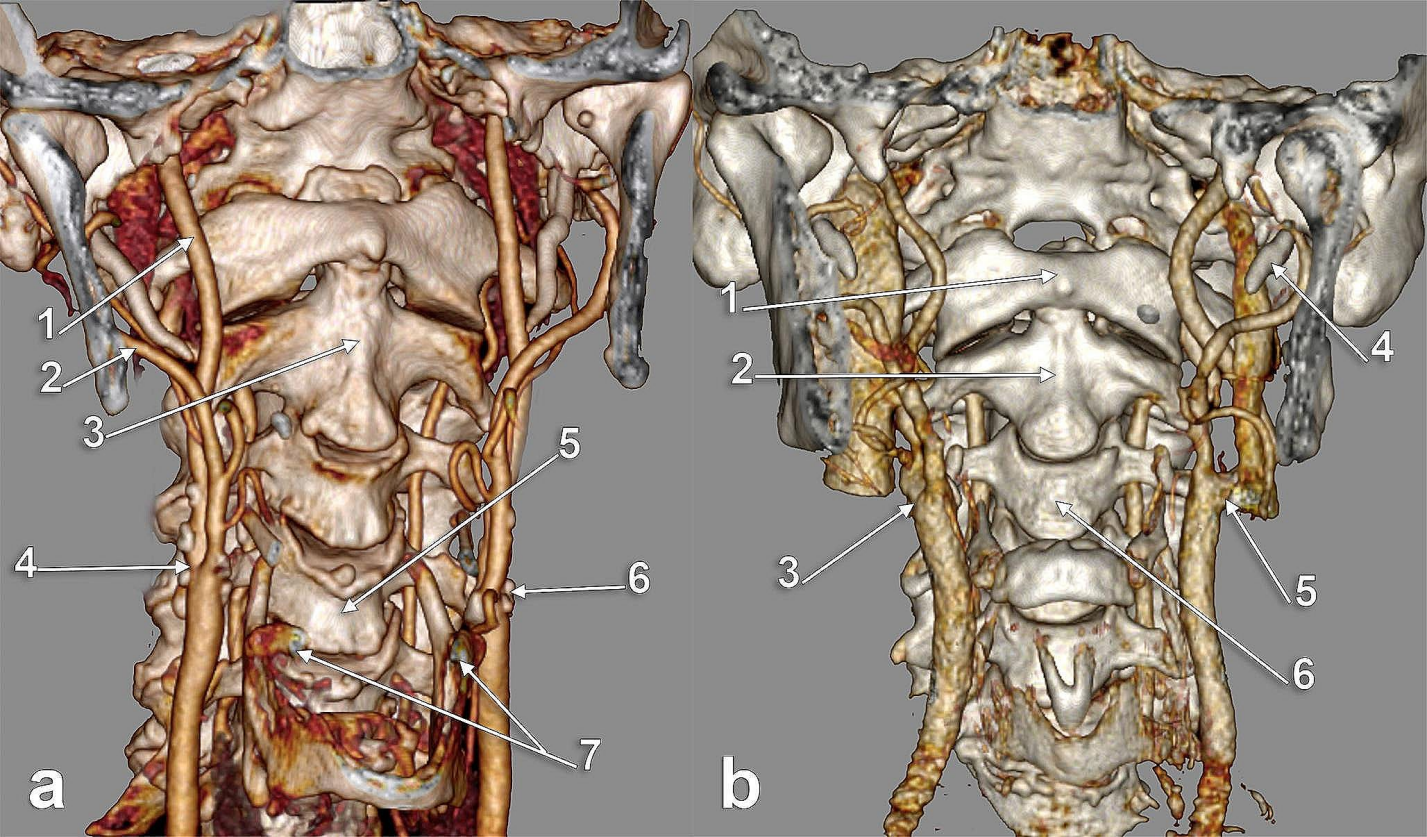
**a**. Carotid bifurcations (CBs) located at C4. Three-dimensional volume rendering. Right anterolateral view. (1) right internal carotid a.; (2) right external carotid a.; (3) axis; (4) right CB; (5) C4 vertebra; (6) left CB; (7) greater horns of hyoid bone. **b.** Carotid bifurcations (CBs) located at C3. Three-dimensional volume rendering. Anterior view. (1) anterior arch of atlas; (2) axis; (3) right CB; (4) styloid process; (5) left CB; 6.C3 vertebra

**Fig. 3:**
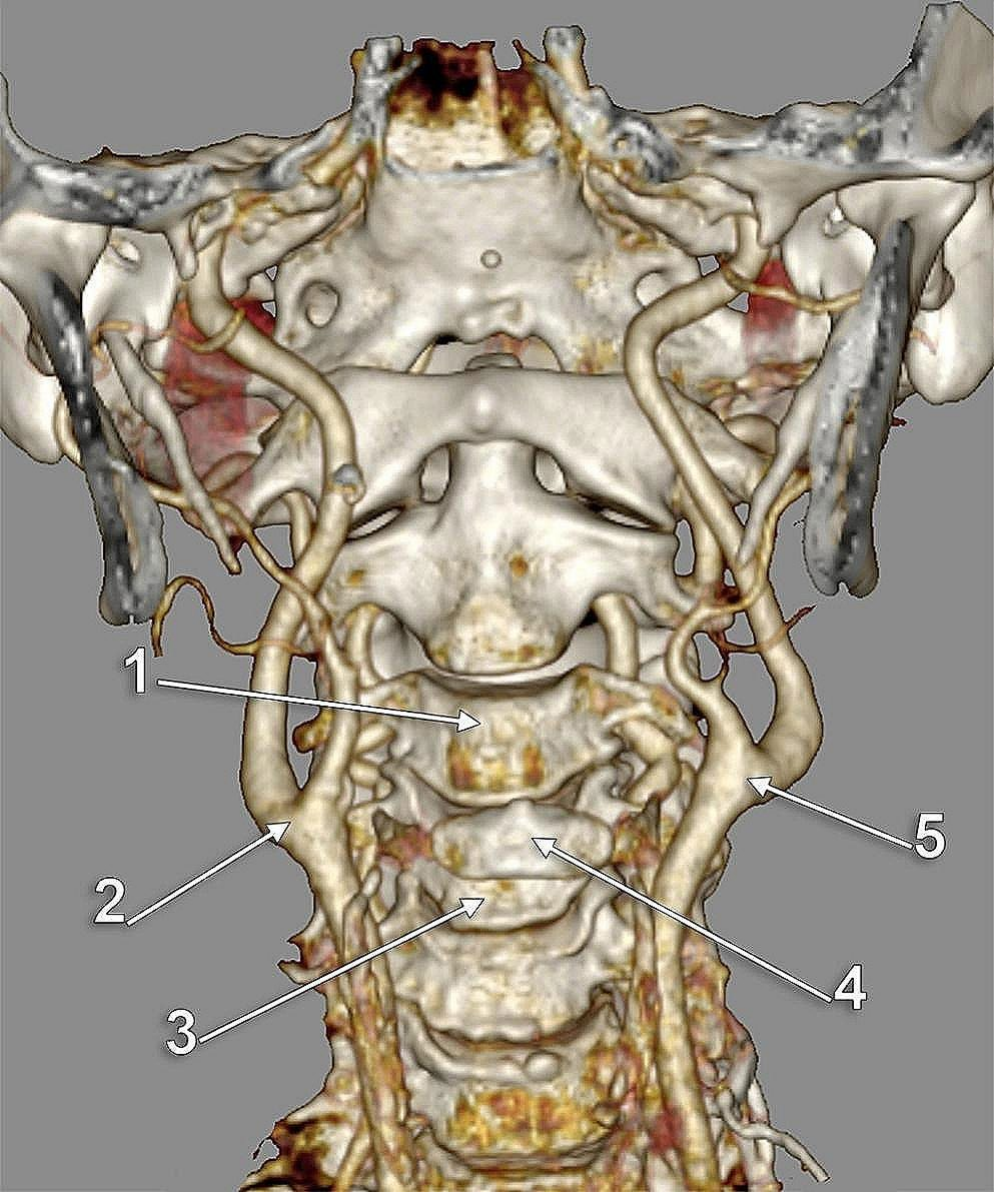
Carotid bifurcations (CBs) located at the C3/C4 intervertebral disc. Three-dimensional volume rendering. Anterior view. (1) C3 vertebra; (2) right CB; (3) C4 vertebra; (4) hyoid body; (5) left CB

The CB was located, on either side of the median plane, from the level of the C2 vertebra (Fig. 1A) to the level of the C5/C6 intervertebral disc (Fig. 1B). In men, the most common CB locations were, in order, at the C4 vertebra (29.07%) (Fig. 2A), C3 vertebra (27.33%) (Fig. 2B) and C3/C4 disc (26.16%) levels (Table 1). In females, the most common locations of CB were, in order, at the C3 vertebra (27.05%), C3/C4 disc (26.23%) (Fig. 3) and C4 vertebra (20.49%) levels (Table 1). In the overall group, the most common locations of CB were, in order, at the C3 vertebra (27.21%), C3/C4 intervertebral disc (26.19%) and C4 vertebra (25.51%) levels (Table 1).

**Table 1:** Vertical level of the carotid bifurcation (CB) in males (172 sides), females (122 sides), and the overall group (294 sides)

CB locations	Men (value/%)	Females (value/%)	Overall group (value/%)
C2 vertebra	2/1.16%	1/0.82%	3/1.02%
C2/C3 disc	10/5.81%	12/9.84%	22/7.48%
C3 vertebra	47/27.33%	33/27.05%	80/27.21%
C3/C4 disc	45/26.16%	32/26.23%	77/26.19%
C4 vertebra	50/29.07%	25/20.49%	75/25.51%
C4/C5 disc	3/1.74%	12/9.84%	15/5.1%
C5 vertebra	15/8.72%	6/4.92%	21/7.14%
C5/C6 disc	0	1/0.82%	1/0.34

**Table 2:** Cases with bilateral asymmetry and symmetry of the carotid bifurcations’ vertebral levels

	Bilateral asymmetry	Bilateral symmetry
**Males**	38/86	48/86
**Females**	33/61	28/61
**TOTAL**	71/147	76/147

**Table 3:** Vertebral (C2 – C5) and intervertebral disc (C2/C3 – C5/C6) levels of the bilaterally symmetrical carotid bifurcations (CBs) in males (N_M_=86), females (N_F_=61) and the overall batch (*N* = 147). NULL: bilaterally asymmetrical CBs.

	NULL	C2	C2/C3 disc	C3	C3/C4 Disc	C4	C4/C5 disc	C5	C5/C6 disc
**Males**	38	0	0	13	13	16	0	6	0
**Females**	33	0	2	9	6	5	4	2	0
**TOTAL**	71	0	2	22	19	21	4	8	0

In men (N_M_=86), the CB showed bilateral symmetry to vertebral landmarks in 55.81%. In women (N_F_=61), bilateral symmetry of CB to vertebral landmarks was recorded in 45.9% of cases. In the general group (*N* = 147), bilateral symmetry of CB to vertebral landmarks was identified in 51.7% (Table 2).

There were no positive cases for bilateral symmetry of the C2 vertebral level of CB. No bilateral symmetry was detected for the C5/C6 level either. In the male subgroup (N_M_=86), bilateral symmetrical CB at C3 was present in 15.12%, C3/C4 in 15.12%, C4 in 18.6% and C5 in 6.98%. In the female subgroup (N_F_=61) bilateral symmetry was at C2/C3 in 3.28%, at C3 in 14.75%, at C3/C4 in 9.84%, at C4 in 8.2%, at C4/C5 in 6.56% and at C5 in 3.28% (Table 3).

Anterior landmarks of the CB

No intrathoracic CB (type 7) was found in the investigated group. Only types 1–6 were identified. In men (172 CBs) type 3 (hyoid level) prevailed (45.35%) (Fig. 5A); type 2 (inter-thyro-hyoid CB) was identified in 29.65%, type 1 (thyroid cartilage level) was present in 8.14%, type 4 (inter-hyo-mandibular level) in 11.63%, type 5 (gonial level) in 2.33% (Fig. 5B) and type 6 (lower cervical level) in 2.91% (Fig. 6A). In women (122 CBs), type 3 (hyoid level) was also prevalent (30.33%); type 1 (CB at the upper border of the thyroid cartilage) was present (Figs. 6B and 7).) in 22.13%, type 2 (inter-thyro-hyoid CB) was identified in 17.21%, type 4 (inter-hyo-mandibular level) in 16.39%, type 5 (gonial level) in 11.48% and type 6 (lower cervical level) in 2.46% (Fig. 7B). In the overall batch (294 CBs), the distribution of the respective types was type 3 (39.12%) – type 2 (24.49%) – type 1 (13.95%) – type 4 (13.61%) – type 5 (6.12%) – type 6 (2.72%) (Table 4).

**Table 4:** Types of vertical levels of the carotid bifurcations as referred to anterior anatomical landmarks

	type 1	type 2	type 3	type 4	type 5	type 6
**Males**	14	51	78	20	4	5
**Females**	27	21	37	20	14	3
**TOTAL**	41	72	115	40	18	8

In men (N_M_=86), the CBs showed bilateral symmetry to anterior cervical landmarks (types 1–6) in 55.81% of cases. In women (N_F_=61), bilateral symmetry of CBs to anterior cervical landmarks was recorded in 65.57% of cases. In the general group (*N* = 147), bilateral symmetry of CBs to anterior cervical landmarks was found in 59.86% (Table 5).

**Table 5:** Cases with bilateral asymmetry and symmetry of the carotid bifurcations’ types (anterior landmarks)

	bilateral asymmetry	bilateral symmetry
**males**	38/86	48/86
**females**	21/61	40/61
**TOTAL**	59/147	88/147

**Fig. 4:**
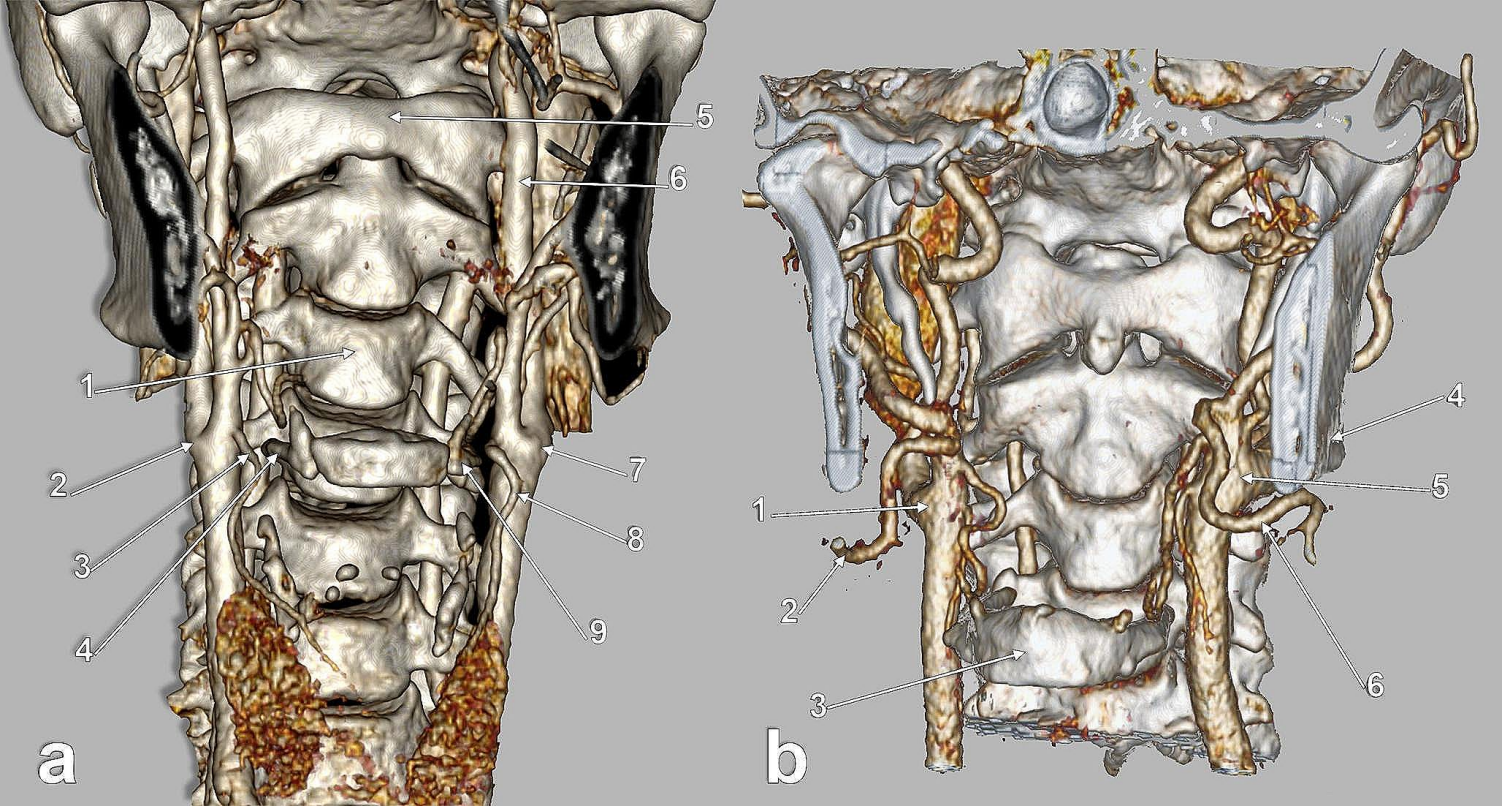
**a.** Discrete vertically asymmetrical carotid bifurcations (CBs), type 3. Three-dimensional volume rendering. Anterior view. (1) C3 vertebra; (2) right CB; (3) right superior thyroid artery; (4) right greater horn of hyoid bone; (5) anterior arch of atlas; (6) left internal carotid artery; (7) left CB; (8) left superior thyroid artery; (9) left greater horn of hyoid bone. **b.** Subgonial carotid bifurcations (type 5) at C2-C3 disc. Three-dimensional volume rendering. Anterior oblique view. (1) right CB; (2) right superior thyroid artery; (3) hyoid bone; (4) left gonial angle; (5) left CB; (6) left superior thyroid artery

In men (N_M_=86) in 44.19%, it was not detected bilateral symmetry for types 1–6; bilateral symmetrical type 1 was present in 2.33%, type 2 in 15.12%, type 3 in 29.07%, type 4 in 5.81%, type 5 in 1.16% and type 6 in 2.33% (Fig. 4B). It was not detected bilateral symmetry for types 1–6 in 34.43% of females (N_F_=61); bilateral symmetrical type 1 was present in 16.39%, type 2 in 9.84%, type 3 in 21.31%, type 4 in 6.56%, and type 5 in 11.48% (Table 6).

**Table 6:** Cases of bilaterally asymmetrical (BA) and symmetrical types 1–6 of the carotid bifurcations in males (N_M_=86), females (N_F_=61) and the overall batch (*N* = 147)

	BA	type 1	type 2	type 3	type 4	type 5	type 6
**males**	38	2	13	25	5	1	2
**females**	21	10	6	13	4	7	0
**TOTAL**	59	12	19	38	9	8	2

**Fig. 5:**
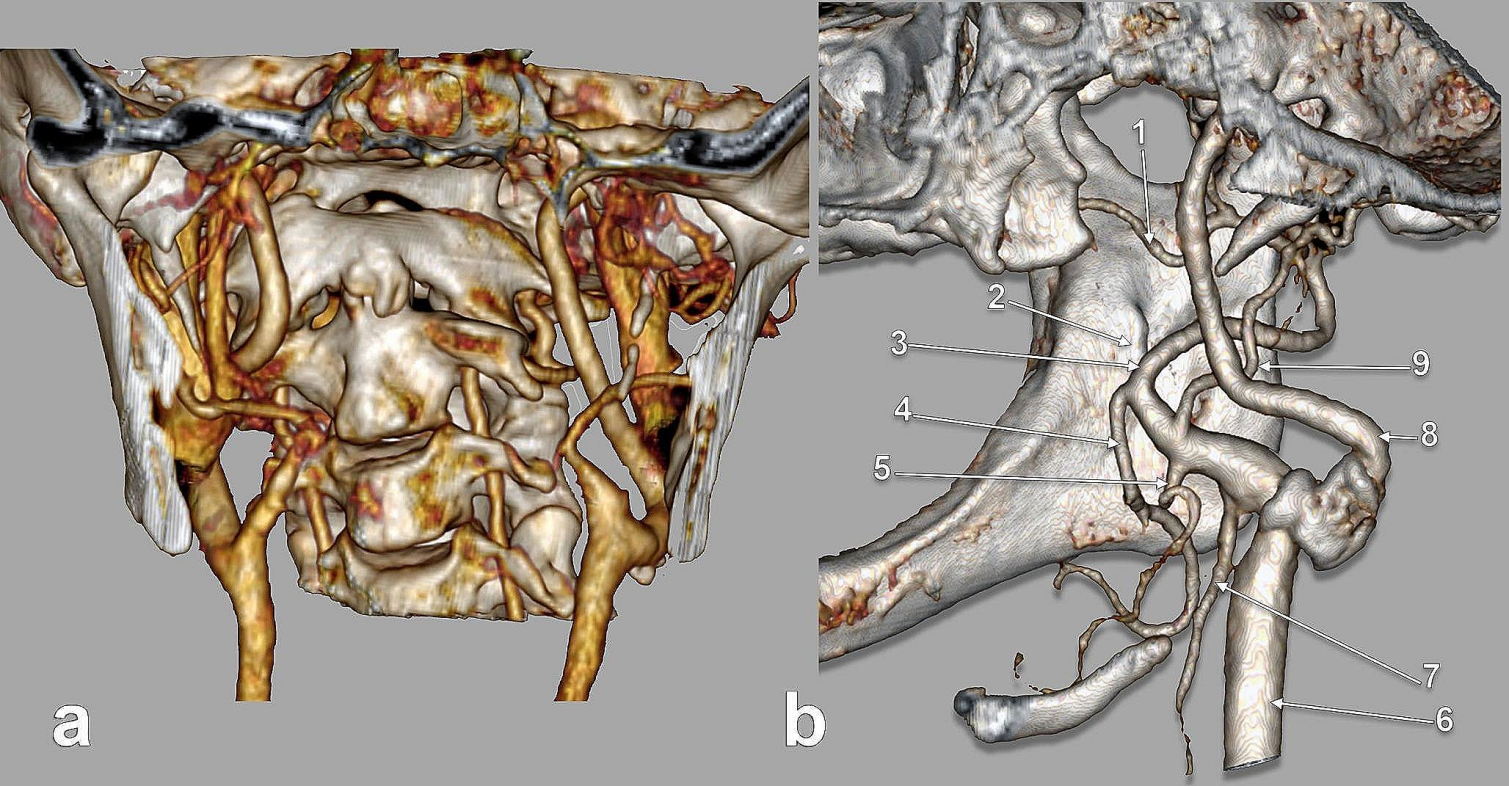
**(a)** Subgonial carotid bifurcations (CBs), type 5. Three-dimensional volume rendering. Anterior view. **(b)** Subgonial right carotid bifurcation (type 5). The external carotid artery sends off collateral branches into the parapharyngeal space. Three-dimensional volume rendering. Medial view. (1) maxillary artery; (2) mandibular lingula; (3) external carotid artery; (4) facial artery; (5) lingual artery; (6) common carotid artery; (7) superior thyroid artery; (8) internal carotid artery; (9) occipital artery

**Fig. 6:**
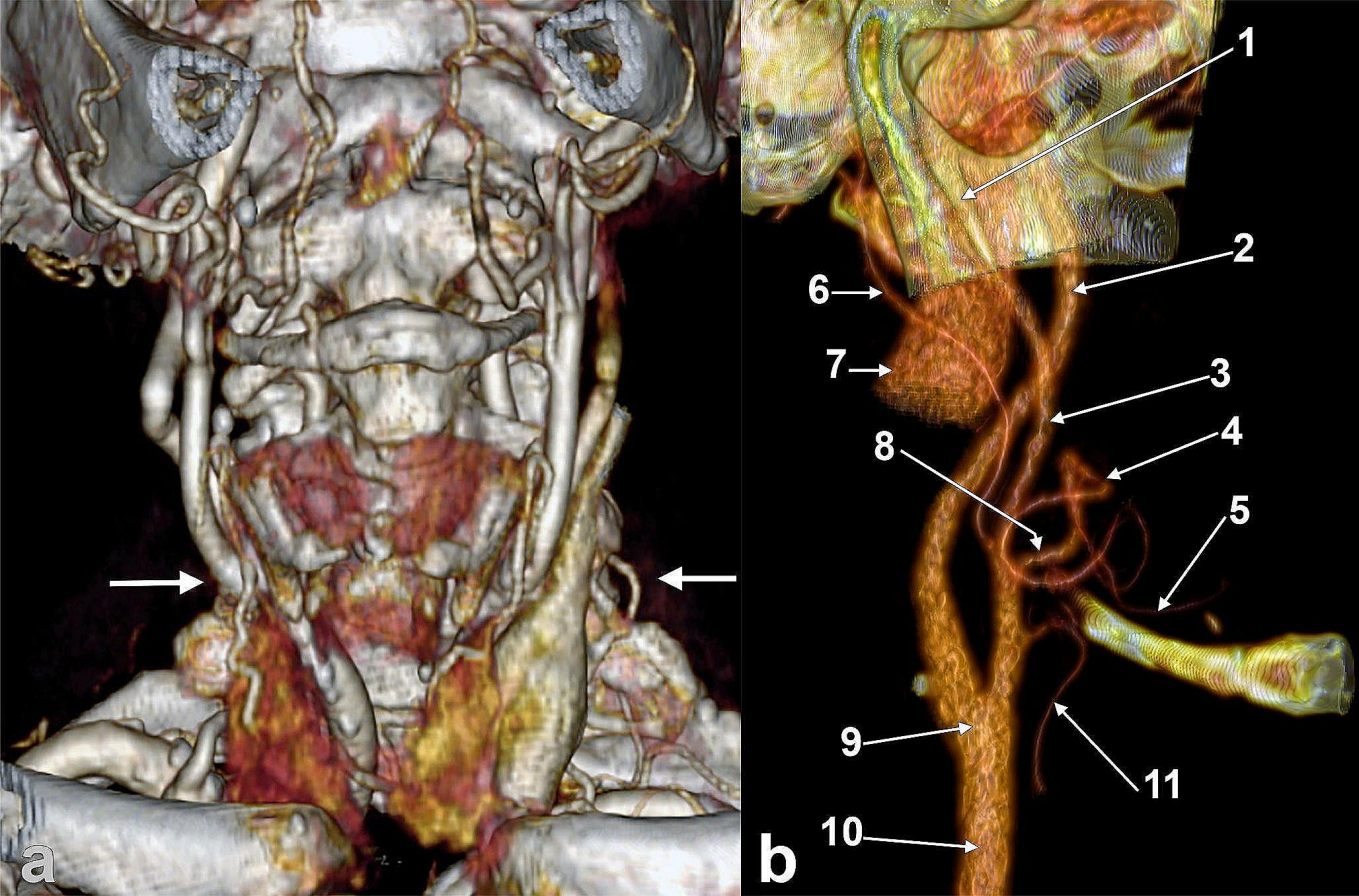
**(a)** Low carotid bifurcations (CBs), bilateral vertical symmetry (arrows). Three-dimensional volume rendering. Anterior view. **(b)** Normal vertical level (type 1) of the right CB. Three-dimensional volume rendering. Right side, lateral view. (1) styloid process; (2) internal carotid artery; (3) external carotid artery; (4) facial artery; (5) lingual artery; (6) occipital artery; (7) internal jugular vein; (8) linguofacial trunk; (9) CB; (10) common carotid artery; 11. superior thyroid artery

**Fig. 7:**
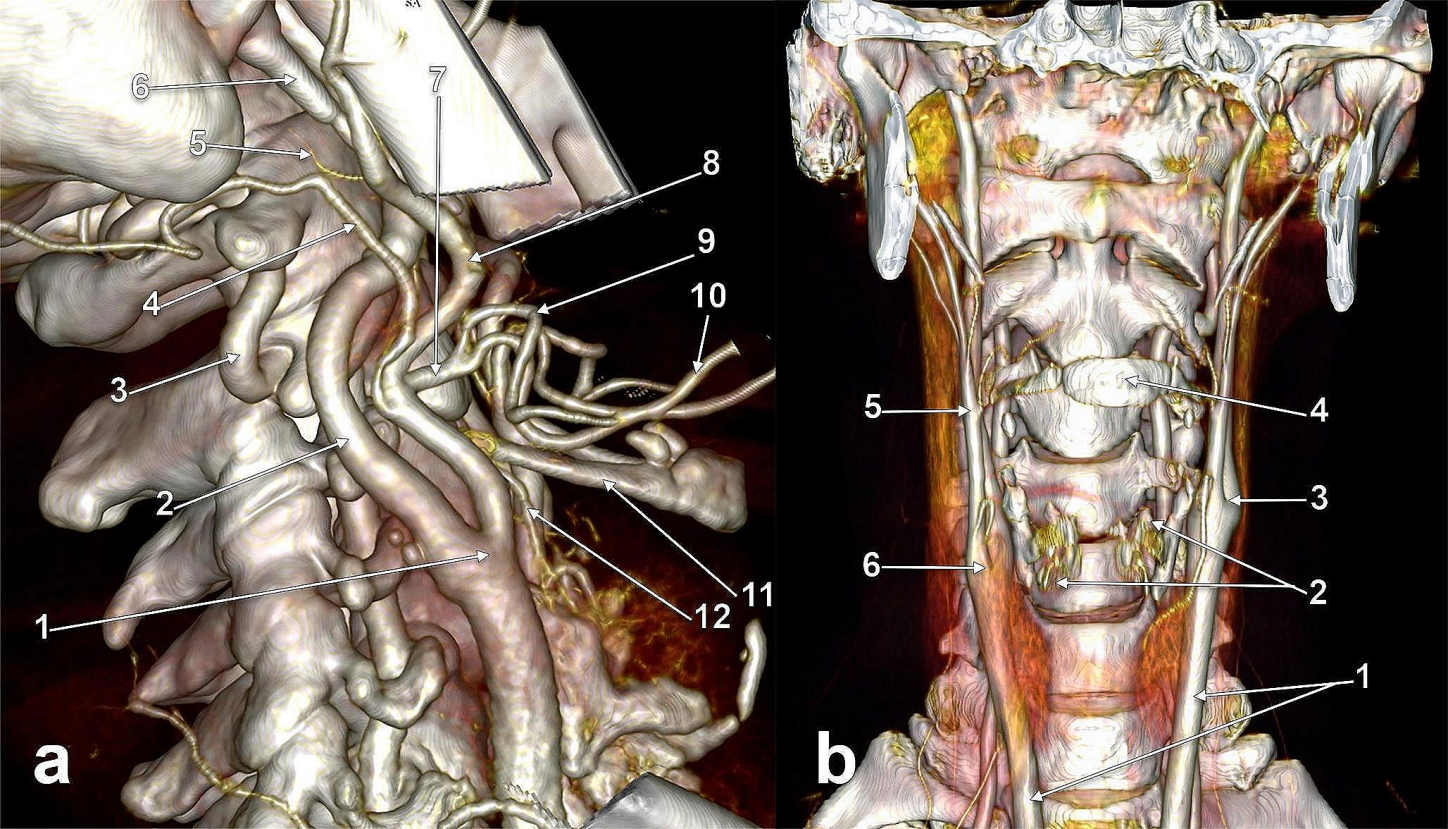
**a.** Normal vertical level of the carotid bifurcation (CB) (type 1). Parallel posterior loops of external and internal carotid arteries. Three-dimensional volume rendering. Infero-lateral view, right side. (1) CB; (2) internal carotid artery; (3) vertebral artery; (4) occipital artery; (5) posterior auricular artery; (6) styloid process; (7) linguofacial trunk; (8) external carotid artery; (9) facial artery; (10) lingual artery; 11. greater horn of hyoid bone; 12. superior thyroid artery. **b.** Bilateral vertical asymmetry of carotid bifurcations (CBs) (right type 6, left type 1). Three-dimensional volume rendering. Anterior view. (1) common carotid arteries; (2) thyroid cartilage; (3) left CB, at the level of the upper margin of the thyroid cartilage; (4) hyoid bone; (5) external carotid artery; (6) right CB, at the level of the lower margin of the thyroid cartilage

Statistical significance

Our research reveals precise and statistically significant differences between sexes and both left and right types 1–6 and vertebral levels of CB.

On the right, types 2 (inter-thyro-hyoid) and 3 (hyoid) are more often encountered in men, while types 1 (thyroid cartilage, typical) and 5 (gonial) – are more often encountered in women (Pearson Chi2 = 12.7, *p* = 0.026). On the left, types 2 (inter-thyro-hyoid) and 3 (hyoid) are more often encountered in men, while in women, types 1–4, ranging from the thyroid to inter-hyo-mandibular level, are very close in range (from 11 to 17 cases). The association is statistically significant (Pearson Chi2 = 17.6, *p* = 0.003).

In men, there is a higher variability of vertebral level of right CB (from C2/C3 to C5, with a higher number of cases at C3 and C4), while in women, most cases had a C3-C4 level. The associations are highly statistically significant (Pearson Chi2 = 29.6, *p* < 0.001). Similarly, on the left, there is a higher variability of vertebral level in men (from C2 to C5/C6) than in women (from C2 to C4). Also, in men, most cases by a wide margin are at the C4 level (30 v. 8 cases), while in women, most cases are at C3 and C3/C4). The association is highly statistically significant (Pearson Chi2 = 27.3, *p* < 0.001).

On the right side, types 1 (thyroid) and 2 (inter-thyro-hyoid) are most often encountered at the C2 level, type 3 (hyoid) – at the C3/C4 level, and type 5 (gonial) – at the C4 level. The associations, which are highly statistically significant (Pearson Chi2 = 147.4, *p* < 0.001), underscore the importance of our findings. On the left side, type 1 is most often associated with a C2/C3 level, type 2 with C3 and C4 levels, and types 3, 4 and 5 with a C3/C4 level. The associations, also highly statistically significant (Pearson Chi2 = 135.5, *p* < 0.001), further validate our research. Thus, the hyoid level could not be associated with a certain vertebral level.

When comparing bilateral vertebral levels of CBs, it was observed that a C2/C3 level of the right CB is usually associated with a C3 level of left CB, and C3, C3/C4, C4 and C5 vertebral levels of the right CB with, respectively, the C3, C3/C4, C4 and C5 vertebral levels of the left CB. These associations suggest an important symmetry (Pearson Chi2 = 336.5, *p* < 0.001).

When comparing bilateral types of anterior cervical landmarks, symmetry was observed in types 1–5, with a highly significant association (Pearson Chi2 = 249.5, *p* < 0.001).

## Discussion

From an anatomical viewpoint, the vertebral level of the CB could supply the general concept of anatomic variability. However, although the height of the CB is referred to vertebrae, this definition is impractical during surgery as neither the patient is placed in an anatomic position nor the vertebrae are accessible [26]. Anterior anatomic landmarks are more practical to locate the CB during surgery [26]. However, although some authors always found the CB posterior inferior to the tip of the greater horn of hyoid bone [20], their finding is contradicted by the present results demonstrating that the greater horn should not be regarded as a unique landmark of the CB.

Different studies assessed the vertical position of the CB bilaterally and found no bilateral asymmetry [17], or the side-to-side differences in the level of CB were not statistically significant [3]. Although not explicitly documented, such vertical asymmetry of the CBs results from the study of McAfee (1953) [24]. In 1979, Smith and Larsen wrote that perusal of the literature had not revealed any report concerning a possible symmetry of the CB, and therefore, they performed their study on 100 angiograms [34]. The authors found that in 22% of the cases, the right CB was superior to the left CB, while 50% had a higher left CB [34]. In a bilateral study of the vertical location of the CB, the right CB was significantly at a higher level than the left one [12]. Lo et al. (2006) found the bilateral asymmetry of the CB in 48% of cases [22]. Woldeyes (2014) found bilaterally asymmetrical CBs in 61.5% of cases [36]. Kurkcuoglu et al. (2015) detected the bilateral asymmetry of CB in 33% of cases [19]. Mompeo and Bajo (2015) found the bilateral asymmetry of the CB in just 10.52% of the 19 studied cases [28]. The present study found bilaterally asymmetrical CBs in 48.3% of cases for the vertebral level and 40.14% of cases for the anterior cervical landmarks. However, it was found here that CB’s bilateral symmetry is equally significant for vertebral levels and anterior cervical landmarks. The anatomical correlation between the vertebral and anterior cervical levels is somewhat unexpected, although different significant associations were established. Other authors did not evaluate the bilateral symmetry of the CB [5].

Different vertical locations of the CB were found in the present study and others [1, 3–5, 9, 11, 12, 14, 17, 19, 21, 24, 25, 27–37]. As presented in Table 7, different studies observed only the vertebral level of the CB; other authors studied the CB in relation to different anterior cervical landmarks, while a few authors observed both vertebral and anterior cervical landmarks. Numerous studies were performed by dissections. Few studies, including the present one, studied > 100 cases. Some of these studies estimated the level of the CB as normal, high or low, just referring the CB to the superior margin of the thyroid cartilage [2, 29].

**Table 7:** Previous studies of the vertical topography of carotid bifurcation determined either vertebral levels, anterior cervical landmarks, or both. Methods and lots are listed. Few studies investigated both anterior and posterior landmarks of the carotid bifurcation. A: angiography; CT: computed tomography; D: dissection

Authors	Method	No. of cases	Vertebral levels of CB	Anterior cervical landmarks of CB
McAfee, 1953 [24]	D	70	-	+
Smith and Larsen, 1979 [34]	A	100	+	-
Lippert and Pabst, 1985 [21]	N/A	N/A	+	-
Toyota et al., 1995 [35]	A	490 cases / 517 arteries	+	-
Lucev et al., 2000 [23]	D	20	-	+
Hayashi, 2005 [17]	D	49	+	-
Zumre et al., 2005 [37]	D	20 fetuses	+	-
Ribeiro et al., 2006 [32]	D	46	+	+
Anu et al., 2007 [4]	D	95	+	-
Ozgur et al., 2008 [29]	D	20	-	+
Anangwe et al., 2008 [3]	D	40	+	-
Mirjalili et al., 2012 [27]	CT	52	+	+
Ambali and Jadhav, 2012 [2]	D	100	-	+
Acar et al., 2013 [1]	CT	100	-	+
Shivaprakash and Vijaykumar, 2014 [33]	D	25	+	-
Radha, 2014 [31]	D	40	-	+
Woldeyes, 2014 [36]	D	13	+	-
McNamara et al., 2015 [25]	CT	86	+	-
Kurkcuoglu et al., 2015 [19]	A	100	+	-
Mompeo and Bajo, 2015 [28]	D	19	-	+
Cappabianca et al., 2016 [7]	CT	253	+	-
Devadas et al., 2018 [14]	D	40	-	+
Arumugam and Subbiah, 2020 [5]	D	25	-	+
Cobiella et al., 2021 [12]	D	**165**	-	+
Chalise et al., 2021 [9]	D	18	+	-
Cihan and Deveci 2022 [11]	CT	**247**	+	+
present study	CT	**147**	+	+

McAfee et al. (1953) performed vertical measurements to detect the CB bilaterally in 70 dissected cadavers [24]. The authors found that CB was in the upper 5th of the neck in 82% of dissections, in an area about 2.5 cm long, measured inferiorly from the inferior margin of the mandible, and in 18% it was located in the upper 2/5 of the neck [24]. The mean distance between the mandible and CB was 2.14 cm [24]. The lowest CB was 7.2 cm superior to the clavicle [24]. McAfee found in 7.1% the CB above the gonial angle. This type 5 variant was found here in 6.12%.

We found by the present study that: (1) the inter-thyro-hyoid and hyoid levels are rather encountered in men, on any side; (2) on the right side, the normal and the gonial types are rather found in women; on the left side in women the CB is rather found in the interval between the upper margin of the thyroid cartilage and the mandible; (3) there is a higher variability of the vertebral level of CB in men; in women, most cases had a C3-C4 level on the right and a C2-C4 level on the left.

The present study found significant associations between certain vertebral levels and anterior cervical landmarks, such as C2-types 1 and 2, C3/C4-type 3, C4-type 5 on the right side and C2/C3-type 1, C3 and C4-type 2, C3/C4-types 3, 4, 5 on the left side. As referred to in typical anatomy, these associations may surprise if one relates the hyoid bone and the thyroid cartilage to a specific vertebra. The vertical position of the laryngeal apparatus and the geometry of the cervical vertebrae should be regarded as variable. Mirjalili’s study [27] also shows that the correspondence between the anatomical position of the anterior cervical landmarks and the vertebral landmarks is not absolute: both the hyoid and the thyroid cartilage can be located anywhere between the C3 vertebra and the C5/C6 disc. However, Mirjalili et al. found no statistically significant differences related to age or sex, and they did not determine the CB topography versus gonial angle [27]. Demirtas et al. recently determined a statistically significant correlation between CB levels and CB angles on both sides: CB angles narrow as bilateral CB levels decrease [13].

Our results converge with the conclusions of a previous study of the anterior and posterior landmarks of the CB. Cihan and Deveci (2022) concluded that estimating the CB’s location according to the gonion and hyoid bone will give a more accurate result [11]. Cobiella et al. (2021) used the body of the hyoid bone level as a single landmark for the CB [12]. The values obtained for the level of the CB ranged from 4 cm below the hyoid body and 2.5 cm above the respective landmark. The authors found no significant differences in relation to the distribution of CB by sex [12].

Hayashi et al. (2005) found the CBs most frequently at the level of the middle 3rd of the C3 vertebra, but the mean position of the CB was located at the lower 3rd of the C3 vertebra [17]. A more recent study of 100 angiograms determined vertebral levels of CB [19]. The highest CB level was at the C2 vertebra, and the lowest was at the C6/C7 intervertebral disc in both sexes [19]. In the general group, CB was found most frequently, in 29% of cases, at the level of the C4/C5 intervertebral disc on the right side of the neck and at the level of the C4 vertebra (26%) on the left side of the neck [19]. A low level of the CB was found just above the C6/C7 intervertebral space by Gulsen et al. (2009) [16]. The intrathoracic CB is extremely rare [18], so it is not surprising that the present study did not find this level.

Another study classified the level of CB into three types: normal, at the level of the upper margin of the thyroid cartilage (60%); high, superior to the thyroid cartilage (40%); and low, not found then [5]. In the present study CBs were found lower than the superior margin of the thyroid cartilage in type 6, in 2.72%.

Anangwe et al. (2008) considered two possibilities of CB: high, above the C3/C4 junction and low, below it [3]. The authors assumed that the C3/C4 junction corresponds to the upper margin of the thyroid cartilage [3]; this vertebral level of the CB is also considered by others [10, 33]. However, they did not perform dissections to validate this topographic correlation; the dissections were limited to the carotid triangle [3], so the vertebral level classification appears speculative. Ferracci et al. (2022) documented that the CB is generally located near the superior border of the thyroid cartilage but in front of the C4–C5 disk [15].

McNamara et al. tested the accuracy of a straight-line distance (SLD) between the skull base and the CB to identify that bifurcation [25]. The authors found that the greater horn of the hyoid had the most significant correlation with the SLD quartile group [25]. According to McNamara et al., a standardised definition of high CB is still lacking as there are different potential approaches to defining it [25]. However, one can classify the level of CB using a purely statistical definition, such as the shortest quartile of a normal distribution, as used by McNamara, or a clinical definition, the level that makes the intervention more difficult for the vascular surgeon and with a higher risk of complications for the patient [8].

Anu et al. (2007) found the CB at C3 in 50% on the right and 55% on the left, at C4 (40% on the right, 35% on the left), at C2 in 10% of cases and at C5 in 1% [4]. Woldeyes (2014) studied by dissection only 13 cadavers and located the CB at C2/C3 (3.85%), C3 (42.31%), C3/C4 (15.38%) and at C4 (38.46%) [36]. Chalise et al. (2021) studied the vertebral level of CB in 18 cadavers; it ranged from C2/C3 to C4 [9]. Cihan and Deveci (2022) found the highest vertebral level of CB in the lower 1/3 of the C2 vertebra and the lowest level in the upper 1/3 of the C6 vertebra [11].

Devadas et al. (2018) followed anterior cervical landmarks in the study of vertical topographic possibilities of CB: superior border of thyroid cartilage (75%), inter-thyro-hyoid level (10%), hyoid level (13.75%) and inter-hyo-mandibular level (1.25%) [14]. These authors did not find the CB below the superior margin of the thyroid cartilage. Their landmarks correspond respectively to levels 1 (13.95%), 2 (24.49%), 3 (39.12%) and 4 + 5 (19.73%) in the present study. The differences in type prevalences between these two studies are consistent.

The limitations of this study are related to the simultaneous variations of the ECA and ICA. Thus, for a specific level of the CB, the branching pattern of the ECA could differ from one case to another. Moreover, a certain topographical level of the CB may not give additional information on the deviated courses of the carotid arteries due to their coiling, kinking, or different axial rotations.

## Conclusions

The vertical topography of the CB is highly variable and has sex-related specificity. This detail should be included in the teaching of anatomy. The right and left types 2 and 3 are rather found in males, and the right types 1 and 5 – in females. The vertebral level of the CB has a higher variability in males, while in females, the CB has usually a C3-C4 level. The hyoid level of the CB should not be associated with a certain vertebral level. The level of the CB is usually bilaterally symmetrical. Therefore, surgeons and interventionists should carefully document the carotid anatomy on a case-by-case basis.

## Data Availability

This study did not create or analyse new data, and data sharing does not apply to this article.
